# Bifunctional Smart Textiles with Simultaneous Motion Monitoring and Thermotherapy for Human Joint Injuries

**DOI:** 10.1002/advs.202305312

**Published:** 2023-11-30

**Authors:** Yingcun Liu, Duo Xu, Can Ge, Chong Gao, Yawen Wei, Ze Chen, Ziyi Su, Keshuai Liu, Weilin Xu, Jian Fang

**Affiliations:** ^1^ College of Textile and Clothing Engineering Soochow University Suzhou 215123 P. R. China; ^2^ State Key Laboratory of New Textile Materials and Advanced Processing Technologies Wuhan Textile University Wuhan 430200 P. R. China

**Keywords:** core‐sheath structure, human health monitoring, integrated sensing yarn, joint injuries, thermotherapy

## Abstract

The motion detection and thermotherapy provides a convenient strategy for the diagnosis and rehabilitation assessment of joint injuries. However, it is still challenging to simultaneously achieve accurate joint motion monitoring and on‐demand thermotherapy. Herein, core‐sheath sensing yarns (CSSYs) is proposed and fabricated for excellent electrical and photothermal heating, which consists of carbon black (CB)‐coated nylon (sheath layer), silver‐plated nylon and elastic spandex yarns (core layer). The CSSYs demonstrates great joule heating performance, which reaches 75 °C at 2 V applied voltage. The good thermal management performance can be well maintained when weaving these yarns into bifunctional smart textile. Further, the optimized double‐ply CSSYs (DPCSSYs) with helically twisted structure possess several appealing sensing performance, including preferable strain sensitivity (0.854), excellent linearity (0.962), and superior durability (over 5000 cycles). The as‐woven bifunctional smart textile can provide instant and convenient thermotherapy to the injured joints, and simultaneously monitor the injury and recovery conditions of the joint. Therefore, the designed bifunctional smart textile can provide a promising route for developing next‐generation healthcare smart textile.

## Graphical abstract

The bifunctional smart textile with accurate motion monitoring and thermotherapy for human joint injuries though the facile fabrication of CSSYs and DPCSSYs. The CSSYs demonstrates great joule heating performance, which reaches 75 °C at 2 V applied voltage. The optimized double‐ply CSSYs (DPCSSYs) with helically twisted structure possess several appealing sensing performance, including preferable strain sensitivity, linearity, and superior durability.

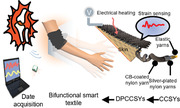

## 1 Introduction

Chronic joint pain has been widely suffered by aged adults with a prevalence of 10‐15% in adults over 60 years of age, it is often caused by long‐term intense labor, which the primary impairments associated with joint injuries include pain, stiffness, joint instability, joint swelling, and muscle weakness.^[^ ^1^, ^2^, ^3^, ^4^ ^]^ At the same time, joint injury is one of the most common forms of sports injury worldwide.^[^ ^5^ ^]^ These symptoms often lead to progressive deterioration of a patient's pre‐injury lifestyle, necessitating long‐term care and treatment strategies. Thermotherapy is an effective, simple, and rapid method for pain relief, commonly used to alleviate tissue and joint pain by promoting vasodilation, reducing muscle spasms, and accelerating cellular activity.^[^ ^6^, ^7^, ^8^, ^9^ ^]^ However, currently available thermotherapy healthcare equipment used in hospital usually have the drawbacks of large size and heavy weight, making them inconvenient for portable use.^[^ ^7^ ^]^ Therefore, there is a pressing need to develop portable and wearable healthcare devices, including electrical heating textiles.

Electrical heating textiles with thermotherapy function have demonstrated vast potential for alleviating joint pain due to their portability, flexibility, conformability, and user‐friendly operation.^[^ ^10^, ^11^ ^]^ Commercial knee heating pads with thermotherapy function are available by incorporating additional electrical heating layer onto conventional compression sleeves. In addition, functional conductive materials, including conducting polymers,^[^ ^12^, ^13^ ^]^ carbon black,^[^ ^14^ ^]^ carbon nanotubes,^[^ ^15^, ^16^, ^17^ ^]^ graphene,^[^ ^18^, ^19^, ^20^ ^]^ and MXene^[^ ^21^, ^22^ ^]^ have been utilized for the fabrication of electroactive textiles with thermotherapy to enhance photo‐to‐thermal or electro‐to‐thermal conversion ability through interfacial polymerization, electroless plating or dip‐coating.^[^ ^23^, ^24^, ^25^ ^]^ For example, Moon et al. fabricated silver nanoparticle ink and directly printed onto medical‐grade adhesive tape to create heating pads.^[^ ^7^ ^]^ Giwon Lee et al. manufactured a stretchable heater for joints thermotherapy, and successfully achieved pain relief.^[^ ^26^ ^]^ Nevertheless, these electrical textiles with thermotherapy lack real‐time motion monitoring function for tracking physical activity of the joint injury patients,^[^ ^27^ ^]^ inevitably decreasing the effectiveness of joint injury rehabilitation. Recently, the burgeoning developments in smart textiles have offered tailored and long‐term treatment of joint injuries under potential risk situations.^[^ ^28^, ^29^ ^]^ In addition, real‐time evaluation of joint injury condition is also required for wearable healthcare devices because an increasing number of symptoms (stiffness, joint instability, joint swelling, etc.) are resulted from inappropriate body activities. Generally, the restricted real‐time motion monitoring and poor thermotherapy functionality have limited the practical application of previously reported smart textiles.^[^ ^30^, ^31^, ^32^ ^]^ However, smart textiles simultaneously satisfying both thermotherapy and real‐time motion monitoring requirements have yet been reported.

**Figure 1:**
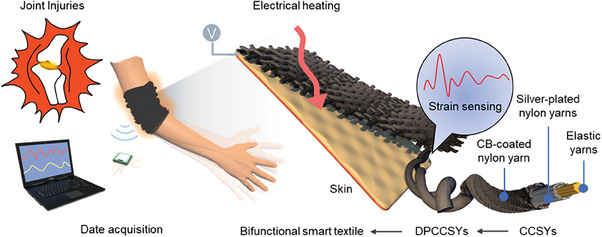
The structural design and functional principle of bifunctional smart textile for motion monitoring and thermotherapy on joint injuries, showing the designable, scalable and facile bifunctional smart textile woven by double‐ply core‐sheath sensing yarns, which consists of CB‐coated nylon, silver‐plated nylon and elastic spandex.

Herein, a new type of bifunctional smart textile has been designed and fabricated using a scalable textile technology for joint injury patients. Through a braiding process, core‐sheath sensing yarns (CSSYs) are firstly fabricated using carbon black (CB)‐coated nylon, silver‐plated nylon and elastic spandex yarns, showing superior electrical and photothermal heating performance. By twisting two CSSYs to form a double‐helix structure, the optimized double‐ply CSSYs (DPCSSYs) can function as capacitive strain sensor, with high strain sensitivity (0.854), outstanding linearity (0.962), and excellent durability (over 5000 cycles). After seamlessly weaving DPCSSYs into flexible fabrics, the resulted bifunctional smart textile integrates capacitive sensing and thermotherapy functions with real‐time healthcare monitoring, photothermal and electrothermal management for joint injury patients (**Figure** **1**). The results have shown the smart textile device can provide efficient thermotherapy to the injured joints, and simultaneously monitor the injury and recovery conditions of the joint. In addition, the bifunctional smart textile demonstrates ideal flexibility and wearability, illustrating the promising potential as practical smart textiles. Therefore, we expect that the bifunctional smart textile will open new doors and provide new insights into personal healthcare monitoring and the thermal treatment of patients with joint injuries.

## 2 Results and Discussion

### 2.1 Fabrication and Characterization of CSSYs

**Figure 2:**
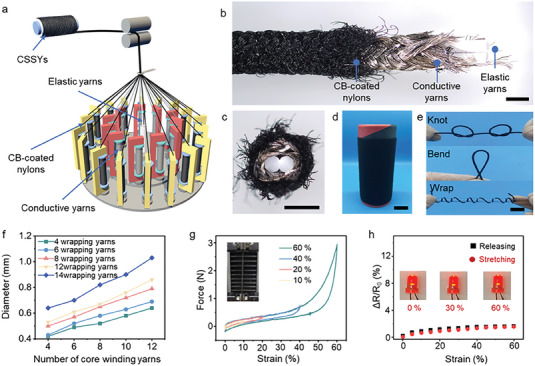
Schematic of the fabrication process and basic performance of CSSYs. a) Fabrication diagram for CSSYs. b) Microscopic image of the structure of CSSYs, scale bars 500 µm. c) Microscopic image of the cross‐section of CSSYs, scale bars, 500 µm. d) A photograph of large‐scale fabrication of CSSYs, scale bars, 3 cm. e) Photograph of CSSYs showing good flexibility for knotting, bending, and wrapping. scale bars, 1 cm. f) Diameter changes of CSSYs under different number of core winding yarns. g) Force‐strain curves of the fabricated CSSYs. h) Relative resistance changes of CSSYs under the different strains.

In this study, a combination of CB‐coated nylon yarns, silver‐plated nylon yarns, and elastic spandex yarns is selected for the proof of concept, and the fabrication process of the braided core‐sheath yarns (denoted as CSSYs) is shown in **Figure** **2**a. A CB coating with polymer binder provides high photothermal conversion efficiency and good insulation, giving CSSYs ideal thermal management performance and excellent dielectric properties. Silver‐plated nylon yarns are used as electrodes and the stretchability of CSSYs are endowed by elastic spandex. The CB‐coated and silver‐plated nylon yarns are wound on the bobbins for braiding. The CB‐coated nylons and silver‐plated nylon bobbins, together with the elastic spandex, are fed into the braiding machine to fabricate braided core‐sheath yarns. As the result of the fabrication process, CSSYs exhibits a typical braided structure, shown in Figure 2b,c. The large‐scale preparation of CSSYs can be achieved by continuously winding nylon yarns and elastic spandex yarns on the cones without length limitation (Figure 2d), proving CSSYs with the potential for industrial production. The CSSYs show good flexibility towards knotting, bending, and wrapping (Figure 2e), showing the suitability for industrial textile processing.

To further investigate the performance of CSSYs, the core‐sheath yarns with different numbers of core‐winding and wrapping yarns are prepared. The results show that the diameter and electrical resistance (Figure 2f; Figure S1, Supporting Information) of CSSYs increase with the increase of the number of winding yarns and wrapping yarns. The mechanical response of CSSYs under dynamic loads is shown in Figure 2g, with a representative engineering force–strain curve at a 60% strain under a force of ≈3 N. The braiding structure of CSSYs provides a wide strain range (Figure S2, Supporting Information), ensuring good stretchability, stability, and durability. The relationship between the CSSYs’ ΔR R_0_^‐1^ and tensile strain is displayed in Figure 2h, showing the resistance change of CSSYs slightly increases with the increase of strain. The brightness of the red LED is unaffected after stretching, which indicates that the resistance of CSSYs is almost unaffected by tensile deformation up to 60%. The stable resistance of CSSYs ensures the subsequent applications.

### 2.2 Thermal Management Performance of CSSYs

**Figure 3:**
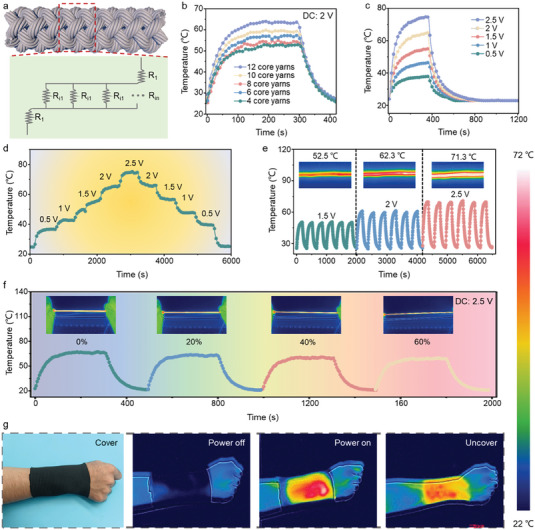
Electrical heating performance of CSSYs. a) The structure and working principle of CSSYs for electrical heating. b) Relative temperature changes of CSSYs under different number of core winding yarns. c) Electrical heating performance of CSSYs at different driving voltages. d) The temperature of CSSYs from 0.5 to 2.5 V and from 2.5 to 0.5 V by adjusting the applied voltage. e) Temperature‐time cyclic curve of CSSYs textile with 1.5 V, 2 V, and 2.5 V input voltage. f) The temperature of CSSYs with 2.5 V input voltage after stretching of 20%, 40%, 60%. g) Digital photo showing the thermotherapy textile made of CSSYs, and IR images of this fabric before and after being applied with a voltage of 2 V.

The synergistic effect of electrothermal and photothermal management can be critical to joint injury treatment.^[^ ^23^, ^24^, ^25^ ^]^ Due to the existence of conductive yarn in the middle layer of CSSYs, the composite yarn can function as an electrical heater.^[^ ^33^ ^]^ As shown in **Figure** **3**a and Figure S1 (Supporting Information), the equivalent circuit diagram of CSSYs exhibits that the electrical resistance decreases with the increased number of conductive nylon yarns due to the structural design of parallel circuit. Finally, CSSYs containing 12 conductive nylon yarns reach a maximum temperature of 62 °C when a voltage of 2 V is applied (Figure 3b), which is consistent with the abovementioned equivalent circuit. We further explore the temperature variation of CSSYs with different applied voltages (Figure 3c), the surface temperature of CSSYs increases from 35 °C at 0.5 V to 76 °C at 2.5 V. Besides, the CSSYs sample shows a quick heating response to the change of applied voltage from 0.5 to 2.5 V (Figure 3d). The CSSYs sample can also exhibit a stable cyclic curve under various applied voltages (Figure 3e) and electrothermal stability for up to 30 min under an applied voltage of 2 V as shown in Figure S3 (Supporting Information), offering great potential for intelligent temperature control. As shown in Figure 3f, the temperature of CSSYs remains almost unchanged with increasing strain rates, indicating that CSSYs enable stable and long‐term use for thermotherapy. Owing to the excellent electrothermal performance, CSSYs can be tailored to continuous weaving process and have been successfully woven into a large piece of fabric (Figure S4, Supporting Information). As depicted in Figure S5 (Supporting Information), CSSYs textile exhibits outstanding water vapor permeability and breathability. Furthermore, even after multiple cycles of stretching and bending deformation, CSSYs textile continues to demonstrate excellent durability and resistance to wear (Figure S6, Supporting Information). As shown in Figure 3g, the infrared images of a piece of thermotherapy textile made of CSSYs under different conditions indicate that the wrist can be uniformly treated with thermotherapy function of the textile.

The textiles with similar parameters are chosen for the test (Table S1, Supporting Information). Furthermore, the mid‐IR spectral reflectivity, transmissivity, and emissivity of different textile fabrics are measured (Figure S7, Supporting Information). The lower mid‐IR spectral emissivity contributes to decreased radiative heat loss, suggesting that the CSSYs textile exhibits reduced thermal radiation loss to the surrounding environment. A self‐designed experimental setup has been utilized to test the passive radiative heating performance of the CSSYs textile (Figure S8a, Supporting Information), wherein the near‐skin temperature covered with CSSYs textile is higher than that of other textile, demonstrating their excellent thermal management performance (Figure S8b, Supporting Information). The CSSYs textile shows a strong optical absorption in the range of 0–2000 nm with superior photo‐to‐thermal conversion ability (Figure S9, Supporting Information). Testing the practical solar heating performance of the CSSYs textile is conducted on a building roof in Wuhan City on November 10, 2022, with real‐time solar intensity shown in Figure S10 (Supporting Information). Various photothermal textiles are exposed outdoors between 9:00 AM and 9:00 PM, and the surface temperatures and infrared images of different textiles are recorded (Figure S11, Supporting Information), and the results indicate that the CSSYs textile exhibits higher temperatures compared with the others. Further testing is conducted to examine the temperature variation of CSSYs textile under 1 V voltage and natural sunlight, revealing its excellent photothermal and electrothermal conversion performance (Figure S12, Supporting Information). The experimental results suggest that the CSSYs textile exhibits excellent photothermal and electrothermal conversion properties, which can possibly be used for the thermotherapy of joint injuries.

### 2.3 Sensing Performance of Double‐ply CSSYs (DPCSSYs)

**Figure 4:**
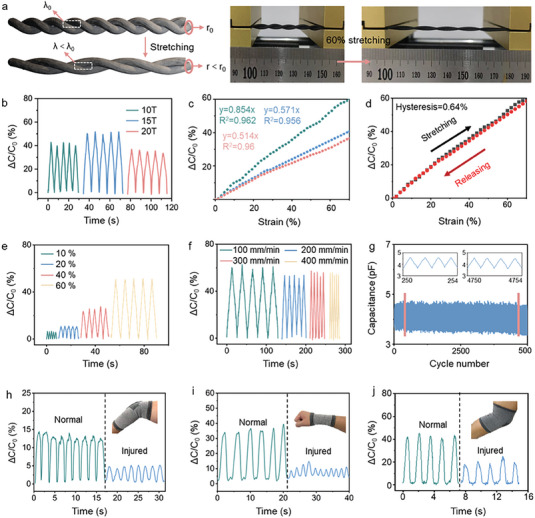
Sensing performance of DPCSSYs. a) Working principle of the DPCSSYs sensor for withstanding strain. b) Relationship between relative capacitance change and twist of DPCSSYs. c) Linear fit curves with different twisting factor of DPCSSYs. d) Hysteresis of DPCSSYs during stretch and release at 70% strain e) Relative capacitance changes of DPCSSYs under the strain of 10, 20, 40, and 60%. f) Relative capacitance changes of DPCSSYs under the speed of 100, 200, 300, and 400 mm min^‐1^. g) Durability of DPCSSYs after 5000 stretch‐release cycles (under the strain of 60% and stretching‐releasing speed of 400 mm min^‐1^). h) Regular capacitance changes during resting state of elbow or elbow injury. i) Regular capacitance changes during resting state of wrist or wrist injury. j) Regular capacitance changes during resting state of knee or knee injury.

The strain sensor of double‐ply CSSYs (DPCSSYs) are manufactured according to the capacitive sensing mechanism, which is produced by twisting two CSSYs into a double‐helix structure (**Figure** **4**a; Figure S13, Supporting Information). The DPCSSYs device's initial capacitance is given by

(1) $$C=\varepsilon_{0}\varepsilon_{r}\frac{S}{\;\lambda }$$

Here *C* is the capacitance, *ε_0_* and *ε_r_* are the dielectric constant of vacuum and the relative permittivity of dielectric media, respectively, *S* represents the area of the electrodes, and *λ* means the thickness of the dielectric layer. According to Equation (1), their sensing mechanism works as follow (Figure 4a): The DPCSSYs device is gradually stretched from the initial diameter r_0_ to a finer diameter *r* (*r<r_0_*) (Figure S14, Supporting Information), while the space between the double‐helical CSSYs is reduced from *λ_0_* to *λ*, resulting increased capacitance. Therefore, the distance between two core‐spun yarns (*λ*) reduces and the area of the electrodes (*S*) increases when the DPCSSYs device is stretched,^[^ ^34^ ^]^ resulting in a significant increase in capacitance. As illustrated in Figure S15 (Supporting Information), the inter stress of DPCSSYs on contact areas during stretched process was simulated by COMSOL software. The simulation results show that as the DPCSSYs stretched, the increased inter stress applied to DPCSSYs causes a reduction in the distance between the two core‐spun yarns and an increase in the electrodes area. The simulation results are almost identical to the calculated results. As shown in Figure S16 (Supporting Information), the stretching and releasing curves of DPCCSYs under different strains and cycles both show highly stable and durable mechanical performance.

The strain‐sensing performance of the DPCSSYs device is measured on the basis of the proposed strain deformation mechanism to demonstrate its potential as a health monitoring device. Figure S17 (Supporting Information) shows the measurement system employed for evaluating the sensing performance of the DPCCSYs. As shown in Figure S18 (Supporting Information), although different the number of core yarns have little impact on the sensing performance of DPCCSYs, the DPCCSYs with 12 core yarns can demonstrate optimal strain‐sensing performance. DPCSSYs subject to a twisting factor of 15 T/10 cm has a good strain‐sensing range and sensitivity with an ideal helical conformation (Figure 4b,c). As illustrated in Figure 4c, the DPCSSYs device with 15 T/10 cm achieves a high strain sensing sensitivity of 0.854 (Table S2, Supporting Information) and excellent linearity of 0.962, respectively, at a maximal strain of up to 70%. The results of the sensitivity results fully show that the 15 T/10 cm DPCCSYs with 15 T/10 cm has excellent strain sensing performance. Furthermore, hysteresis behavior of the yarn device illustrates that DPCSSYs only possess a negligible hysteresis (0.64%) during stretch‐release cycles at 70% tensile strain (Figure 4d). Notably, DPCSSYs still exhibit low hysteresis (0.94%) even after 5000 loading‐unloading cycles (Figure S19, Supporting Information). We further investigate the response of DPCSSYs to cyclic tensile strains of 10%, 20%, 40%, and 60% at a frequency of 0.5 Hz (Figure 4e). As shown in Figure S20, the DPCSSYs shows periodic changes in *ΔC C_0_* ^‐1^ under the lower strain loads, especially at 0.1% strain, verifying its superior ultralow strain detection limit. The *ΔC C_0_* ^‐1^ of the device increases from 5% to 50%, which may be a result of the increased contact area as the tensile strain increases.^[^ ^35^ ^]^ The relative capacitance changes of DPCSSYs at a strain of 60% and speeds of 100, 200, 300, and 400 mm min^‐1^ are shown in Figure 4f. As depicted in Figure S21 (Supporting Information), DPCSSYs exhibit a rapid electric response with the capacitance change completes within 80 ms. The deformation detection capability can be seen that the *ΔC C_0_* ^‐1^ increases linearly with stretching and then maintains a steady peak value without any overshoot and drift, demonstrating the superiors of low creep and stable response (Figure S22, Supporting Information).^[^ ^36^, ^37^ ^]^ The frequency responses minimally affect the relative capacitance change, revealing stable durability. To confirm ideal stability and durability of the DPCSSYs device, 5000 loading–unloading cycles at 60% strain are performed (Figure 4g), demonstrating favorable electromechanical stability and durability. We further test the durability of DPCCSYs, the sensing performance of DPCCSYs remain almost constant of several washing and friction, because of core‐sheath design of CCSYs (Figure S23, Supporting Information). The above results clearly confirm that the mechanical stability and durability of DPCSSYs make it appropriate for practical sensing applications.

A joint is at risk of injury during excessive exercise or exercising with incorrect postures.^[^ ^38^, ^39^ ^]^ To evaluate the motion monitoring performance of the DPCSSYs device, we attach it to various joints on human body (Figure S24, Supporting Information) and detect different motions of the participants. We predict that the motions of normal and injured joints can be distinguished by the strain sensor via the capacitance signal. The experimental results have demonstrated that the intensity and shape of the sensor signals can be clearly differentiated from the motions of normal and injured joints. When the participants bend their injured and healthy elbows (Figure 4h), DPCSSYs generate signals of different intensities, because the injured joint is much stiffer and produces smaller stain level on the sensing element. Similarly, the DPCSSYs devices are able to differentiate healthy and injure wrist joint motions (Figure 4i,j), highlighting its ability to monitor joint injury conditions. The joint monitoring system can accurately detect if the joint is injured or at normal resting state, demonstrating the utility and convenience in joint condition monitoring.

### 2.4 Bifunctional Smart Textile with Motion Monitoring and Thermotherapy Functions

**Figure 5:**
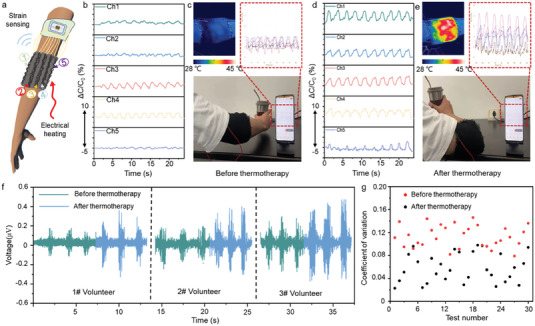
Potential application of the bifunctional smart textile. a) Schematic diagram showing the creation of the bifunctional smart textile. b) Real‐time monitoring of physiological signal changes on the elbow before thermotherapy. c) The thermal image of elbow and the real‐time elbow motion signals before thermotherapy. d) Real‐time monitoring of physiological signal changes on the elbow after thermotherapy. e) The thermal image of elbow and the real‐time elbow motion signals after thermotherapy. f) EMG signals with elbow motions with different volunteers before and after thermotherapy. g) Coefficient of variation of EMG signal before and after thermotherapy.

We further explore the application of our flexible sensor through the fabrication of a bifunctional smart textile with five channels of DPCSSYs devices, as shown in **Figure** **5**a. Based on structural design, CCSYs and DPCCSYs are introduced into the looms to fabricate bifunctional smart textile, integrating with unique functions (Figure S25a,b, Supporting Information). As illustrated in the diagram, the bifunctional smart textile consisting of DPCCSYs and CCSYs (Figure S25c, Supporting Information), which exhibits outstanding thermal management performance and exceptional sensing performance. When wearing these textiles for personal health management, the localized DPCCSYs can show five signals of capacitance change in real time and multiple CCSYs are capable of applying thermotherapy to specific joint areas for pain relief. As shown in Figure 5b, limited range of elbow motion is observed prior to thermotherapy due to the presence of joint pain, resulting in lower electrical signals. Figure 5c depicts the bifunctional smart textile detecting elbow movement through five channels before thermotherapy and wirelessly transmitting it to a smart phone. The IR thermal image in Figure 5c shows that the surface temperature is 28 °C on the bifunctional smart textile before thermotherapy. As show in Figure 5d,e, after a 30‐minute thermotherapy session at 45 °C, the thermotherapy loosens stiff muscles and tissues around the joint injury area, leading to an increased range of motion.^[^ ^6^, ^26^, ^40^, ^41^, ^42^ ^]^ Movie S1 (Supporting Information) demonstrates the bifunctional smart textile monitors elbow movement while simultaneously applying thermotherapy. As shown in Figure S26a (Supporting Information), limited range of elbow motion due to the presence of joint pain, which can be reflected from the weak electrical signals. After a 30‐minute thermotherapy session at 45 °C, the increased range of elbow motion leads to the electrical signal with a noticeable augmentation, providing solid evidence that the reduction in elbow pain after thermotherapy resulted in an increased range of motion (Figure S26b, Supporting Information).

To ascertain the impact of thermotherapy on the elbow joint, the electromyography (EMG) signals around the injured elbow are also recorded. Figure 5f illustrates the EMG signals of the elbow muscles before and after the thermotherapy. Following the 30‐minute thermotherapy session, increased EMG signals can be recorded on all three volunteers, suggesting an expansion in the range of body motion. The EMG signals and real‐time monitoring of physiological signals exhibit consistency before and after the thermotherapy. Monitoring EMG signals around various body joints before and after the thermotherapy reveals that the thermotherapy can effectively alleviate joint pain (Figure S27, Supporting Information). The coefficient of variation of EMG signals is typically defined as the ratio of the standard deviation to the mean, it typically denotes the dispersion of data, with a smaller coefficient of variation indicating stabler thermotherapy effect, demonstrating the feasibility of the thermodynamic effects from bifunctional smart textile.^[^ ^43^ ^]^ The coefficient of variation after the thermotherapy is greater than that before the thermotherapy, indicating that the thermotherapy provided by the bifunctional smart textile helps to alleviate the joint pain (Figure 5g). In comparison to the previous reports on stretchable heating textiles,^[^ ^44^, ^45^, ^46^, ^47^ ^]^ the bifunctional smart textile possess superior comfort, good flexibility and breathability. In addition, it can achieve simultaneous thermotherapy and motion detection, using yarn structured devices fabricated through continuous and scalable processes. These results illustrate that our bifunctional smart textile can possess both thermotherapy and healthcare monitoring capabilities, which can be utilized to manufacture next‐generation personal healthcare monitoring and thermal treatment devices.

## 3 Conclusion

In summary, we have demonstrated bifunctional smart textile with accurate motion monitoring and thermotherapy for human joint injuries though the facile fabrication of CSSYs and DPCSSYs. The CSSYs device shows superior thermal management performance and have a higher temperature (≈10 °C) than the ambient temperature when being applied with voltage of 1 V. By twisting two CSSYs fabricates DPCSSYs, the assembled DPCSSYs achieves high sensitivity (0.854), outstanding linearity (0.962), and excellent durability (over 5000 cycles), which can be utilized to sensitively monitor human joint. Using seamlessly weaving process to manufacture CSSYs and DPCSSYs into bifunctional smart textile, it can provide thermotherapy for human joint while monitoring injury conditions of the joint. After comparing the signal variations between capacitance strain‐sensing and EMG on joint region during thermotherapy, the results demonstrate that bifunctional smart textile enables effective healthcare in treating and monitoring joint injuries for practical applications. This work offers a promising approach for the advancement of multi‐functional smart textile aimed in facilitating rehabilitation treatment.

## Conflict of Interest

The authors declare no conflict of interest.

## Author Contributions

Y.L. and D.X. contributed equally to this work. W.X., and J.F. conceived and planned this research. Y.L., D.X., C.G., and Y.W. performed the experiments. Y.L., D.X., C.G., and Z.C. performed the thermal analysis. Y.L., D.X., Z.S., C.G., and K.L. organized the data and wrote the manuscript. All authors discussed the results and approved the final version of the manuscript.

## Supporting information

Supporting Information

Supplemental Movie 1

## Data Availability

The data that support the findings of this study are available from the corresponding author upon reasonable request.
