# Supplementary material for: Bifunctional Smart Textiles with Simultaneous Motion Monitoring and Thermotherapy for Human Joint Injuries
Source: Adv Sci (Weinh). 2023 Nov 30;11(4):2305312. doi: 10.1002/advs.202305312 (PMC10811511; doi:10.1002/advs.202305312)
Supplement: Supplementary file 1 — Supporting Information [file ADVS-11-2305312-s001.pdf]

ADVANCED SCIENCE
Open Access

Supporting Information

for *Adv. Sci.*, DOI 10.1002/advs.202305312

Bifunctional Smart Textiles with Simultaneous Motion Monitoring and Thermotherapy for Human Joint Injuries

Yingcun Liu, Duo Xu, Can Ge, Chong Gao, Yawen Wei, Ze Chen, Ziyi Su, Keshuai Liu, Weilin Xu and Jian Fang**

Supporting Information

Bifunctional Smart Textiles with Simultaneous Motion Monitoring and Thermotherapy for Human Joint Injuries

Yingcun Liu†, Duo Xu†, Can Ge, Chong Gao, Yawen Wei, Ze Chen, Ziyi Su, Keshuai Liu, Weilin Xu*, Jian Fang*

This PDF file includes:

Supplementary Materials

Movie S1.

The bifunctional smart textiles monitoring of elbow movements before and after thermotherapy.
Supplementary Text S1.

Figure S1 to S27.

Table S1 to S2.

Movie S1.

Experiment section

Materials and methods: Highly conductive silver-plated nylon yarns (60 D, approximately 40 Ω m^{-1}) were purchased from Kazhtex Silver Tech Co. Ltd., China. Elastic yarn (Spandex, 420 D) and CB-coated nylon yarn (100 %, 40 D) were obtained from Hubei Chemical Fiber Co., Ltd., China. Commercial Mylar blanket (~50 μm thickness, 40 g m^{-2}) and graphene oxide (GO) fabric (~350 μm thickness, 200 g m^{-2}) samples were purchased from online stores.

Fabrication of CSSYs: CSSYs were manufactured using a high-speed rope-braiding machine. Silver-plated and CB-coated nylon yarns were sequentially wound onto the bobbins and secured to the inside and outside of yarn discs, respectively. The central elastic yarn was wound on a constant bobbin and fed via a pre-tension device, whereas the CSSYs samples produced using silver-plated nylons and CB-coated nylons were hierarchically interlocked on the elastic yarn as the disc rotated during the operation of the high-speed braiding machine with a braiding speed of 15 rpm, winding speed of 2 m min^{-1}, and braided pitch of 80 mm. Reciprocating movement from one disk to the other was beneficial for the interconnection between the silver-plated nylons and CB-coated nylons to form CSSYs. A continuous supply of yarn was produced by rotating the spool

on the spindles, the number of spindles required for winding the yarn depended on the number of feeding spindles.

Fabrication of DPCSSYs: Two strands of CSSYs were coiled in parallel on a tube and twisted to form a DPCSSYs with a helical conformation. The density of the DPCSSYs could be effectively controlled by varying the fabrication parameters (twisting factor and winding speed).

Fabrication of CSSYs textile: The CSSYs textile, using the SGA598-SD semiautomatic weaving loom, was woven with DPCSSYs as the weft yarn and CB-coated nylon as the warp yarn.

Characterizations: The morphology of the CSSYs were observed using a scanning electron microscope (FEI XL30, Sirion SEM (5 kV)) and a light microscope (RH2000, HIROX, Japan). An INSTRON 5943 strength meter was used to measure the tensile properties of CSSYs. The relative capacitance changes in the CSSYs were measured using a capacitance meter (TH2638, Tong Hui Ltd., Changzhou City, China). The IR reflectivity and transmittance were measured using an FTIR spectrometer (Spotlight 200i, PerkinElmer) equipped with an infrared integrating sphere. Fourier transform infrared (FTIR) spectra were obtained using an FTIR instrument (Nicolet iS50, Thermo Fisher). UV-vis-NIR reflectivity and transmissivity were measured using a UV-vis-NIR spectrometer (UV-3600Plus, SHIMADZE) accompanied by an integrating sphere attachment. A passive radiative heating test was performed indoors; a heating plate was placed onto insulating foam, and the DC power (PPS3005S ATTEN) supply was connected to the heating plate at a power density of 70 W m^{-2} to keep the temperature of the heating plate around 36 °C. The heating plate was then wrapped in the CSSYs textiles, and a thermocouple sensor (TS-08A SHSIWI) was used to measure the skin surface temperature under the textiles. Photothermal conversion tests were conducted outdoors in Wuhan, China. For the outdoor solar heating test, a solar power meter (TES1333R) was used to monitor the real-time solar intensity. A piece of thermally conductive tape was attached to insulating foam to simulate human skin, with a thermocouple sensor fixed onto the thermally conductive tape. The surface of the thermally conductive tape was covered with CSSYs textile. The insulating foam was attached to a melamine foam board for heat insulation. Joule heating performance was evaluated by applying DC voltage at both ends of the CSSYs. A thermocouple sensor (UT3208, UNI-T) was used to measure the temperature of the CSSYs. A G571 air permeability tester (Standard International Group (HK)

Limited, China) was used to test the air permeability of the textile samples with a testing area and pressure of 20 cm^2 and 200 Pa, respectively, according to GT/T 5453. The water vapor transmission rate of the various stretching textiles was tested by the upright cup method in which the size sample was 25 cm^2, temperature was 38±0.6 °C, and relative humidity was 90±2 % based on ASTM E398. A thermocouple sensor (UT3208, UNI-T) was used to record the temperatures of CSSYs textiles artificial skin and the human body, respectively. The Noraxon Ultium EMG was employed to monitor electromyographic (EMG) signals before and after thermotherapy. Human participants gave permission for the collected data (physiological signal detection and motion tracking) to be used in this study via consent forms. The experimental protocols were approved by the Research Ethics Committee of Soochow University (grant number: 52173059) and informed consent form with signature was obtained from the volunteer for the human activity experiments.

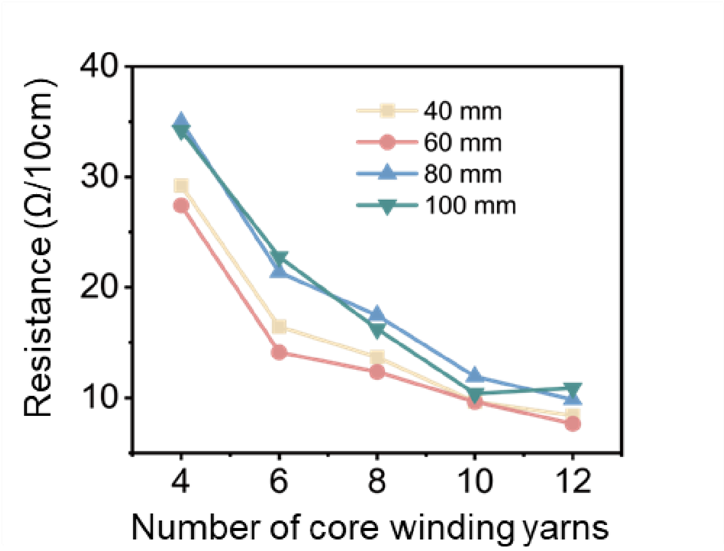

Figure S1. The relationship between the electrical resistance of CSSYs and the number of core winding yarns.

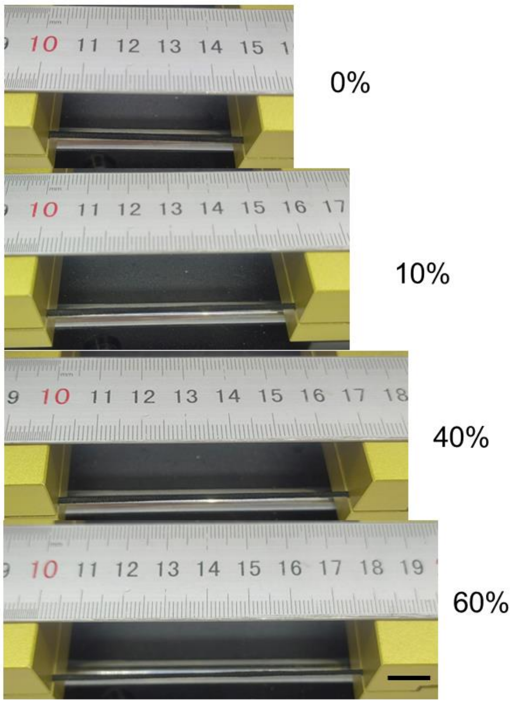

Figure S2. Stretching of CSSYs at different strain levels, scale bar 1 cm.

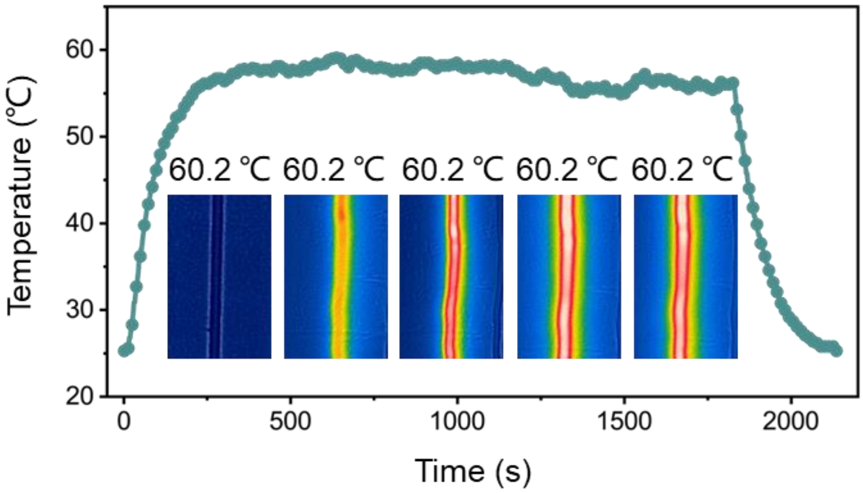

Figure S3. The temperature curve and infrared image of CSSYs under a 2 V input voltage.

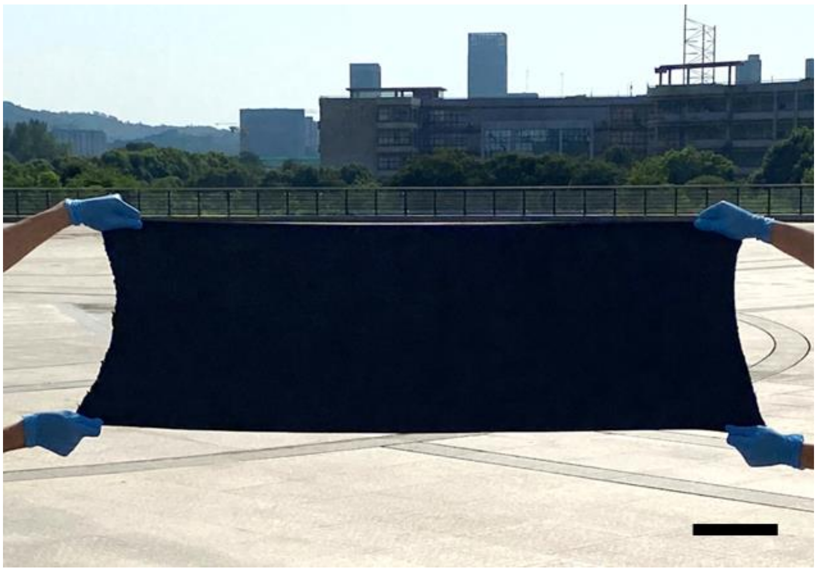

Figure S4. A large piece of fabric woven by CSSYs, scale bar 10 cm.

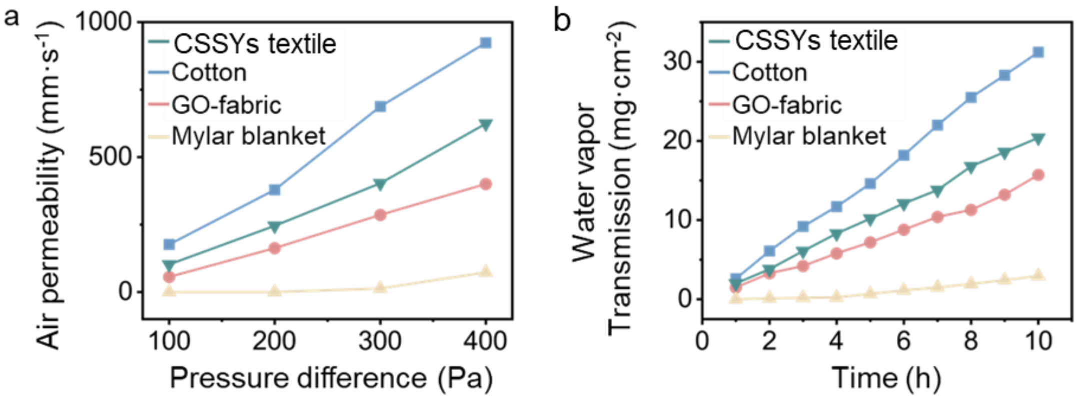

Figure S5. (a) Water vapor transmission rate and (b) air permeability of the CSSYs textile, cotton textile, GO-fabric and Mylar blanket.

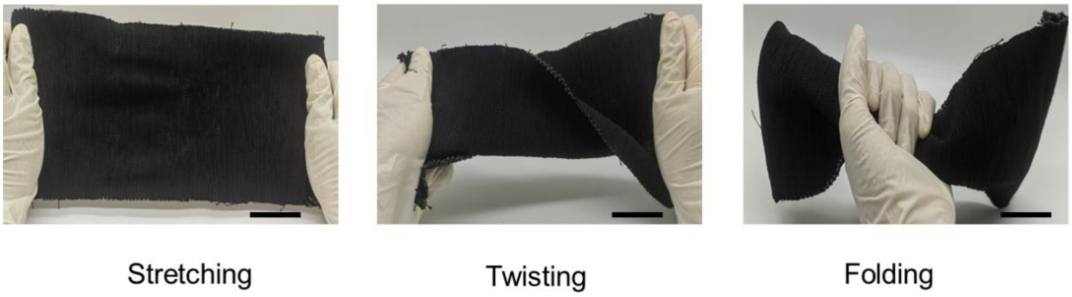

Figure S6. The optical images demonstrate the flexibility, scalability, and stretchability of the CSSYs textile, scale bar 1 cm.

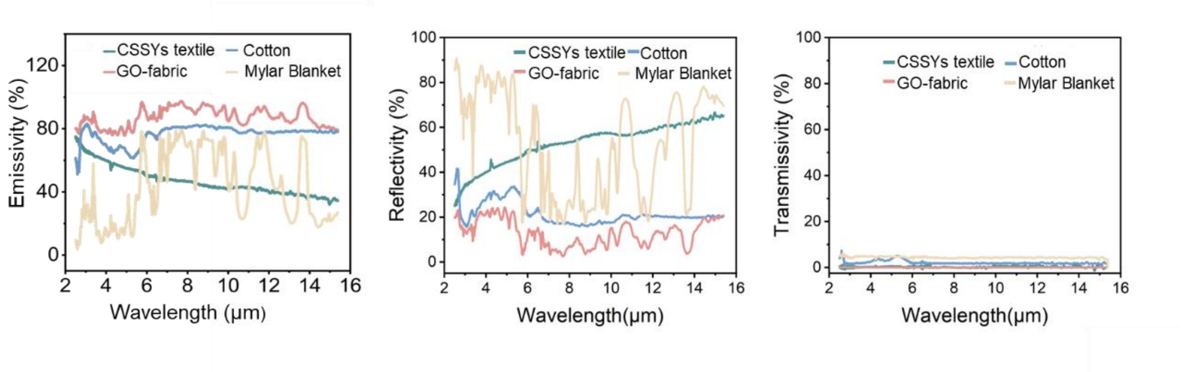

Figure S7. Measured (a) emissivity, (b) reflectivity and (c) transmissivity of CSSYs textile, cotton, GO-fabric and Mylar blanket from ultraviolet to near-infrared wavelength.

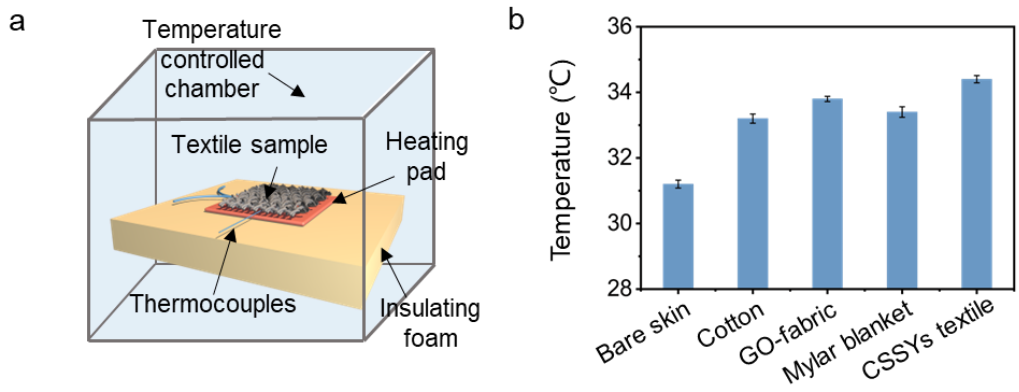

Figure S8. (a) Schematic of experimental setup for indoor passive radiative heating measurement. (b) Temperatures of the artificial skin covered with different textiles in an indoor environment with environmental temperature controlled at 16 ± 0.5 °C.

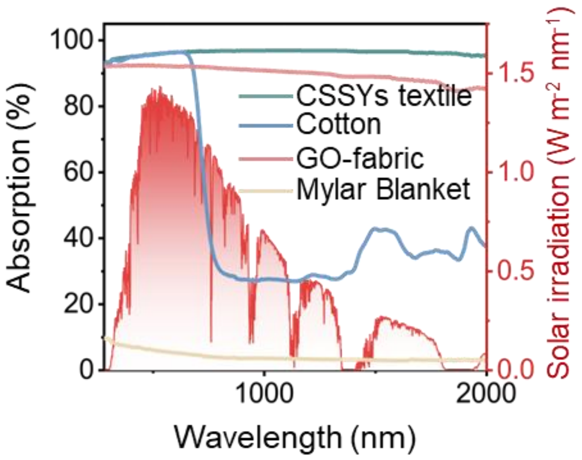

Figure S9. Absorptivity of CSSYs textile, cotton textile, GO-fabric, and Mylar blanket from ultraviolet to near-infrared wavelengths. The light red area represents the solar spectral irradiance.

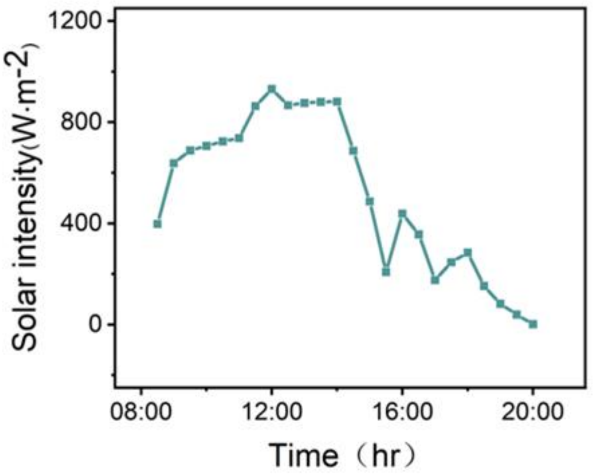

Figure S10. The real-time solar intensity on the roof in Wuhan on November 10, 2022.

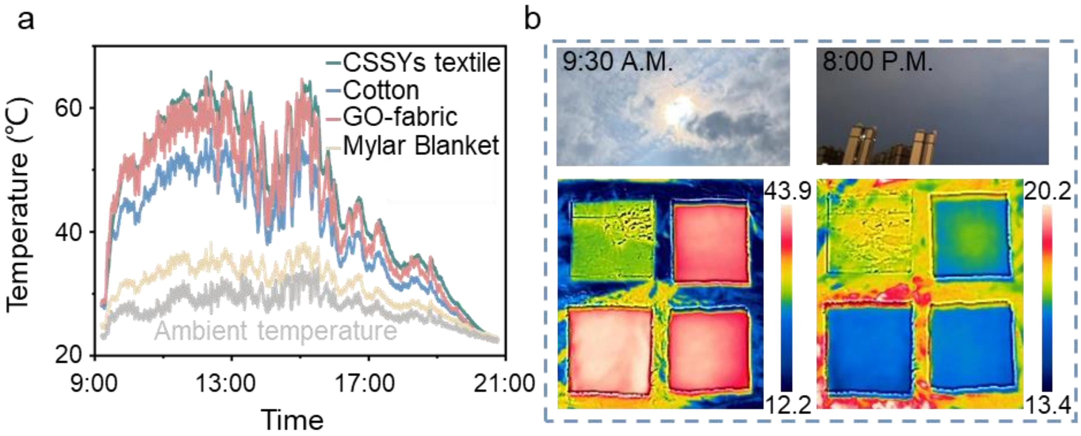

Figure S11. (a) Temperature of artificial skin covered with different textiles on a sunny day. (b) Digital images of real-time weather conditions at 9:00 AM and 9:00 PM on the sunny day and the corresponding thermal infrared images of different textiles.

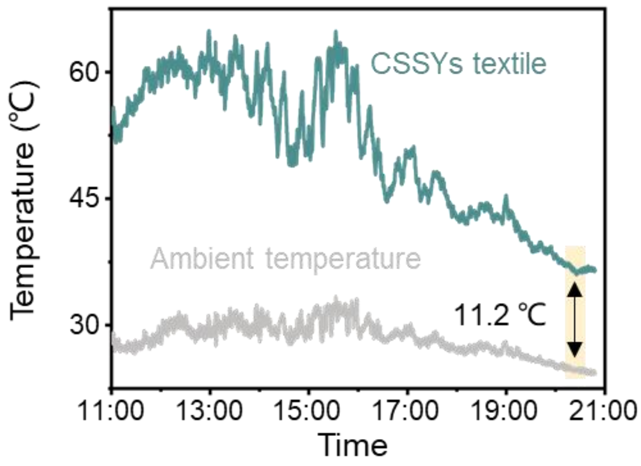

Figure S12. Temperature curve of heating under 1 V from 11:00 AM and 9:00 PM in an outdoor environment.

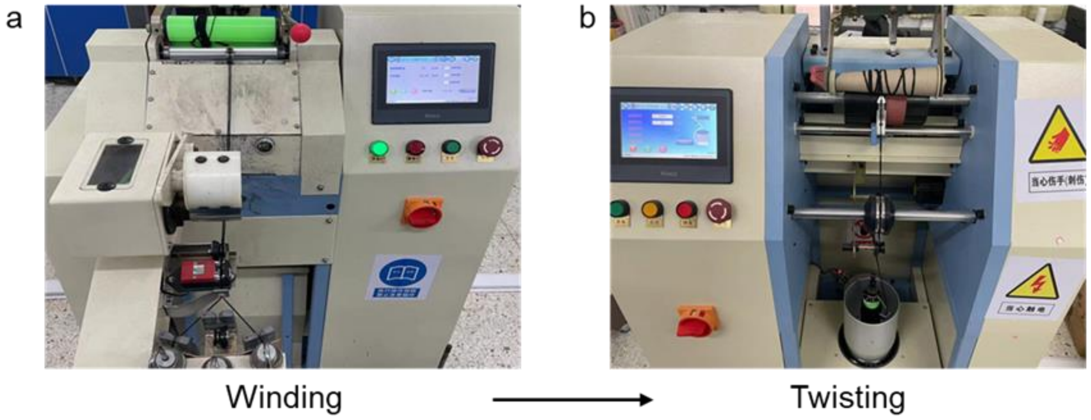

Figure S13. DPCSSYs preparation process. (a) Winding process of two-strand CSSYs. (b) Twisting process of CSSYs.

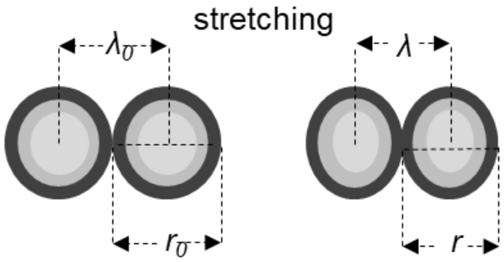

Figure S14. Schematic cross-section illustration of DPCCSYs before and after stretching.

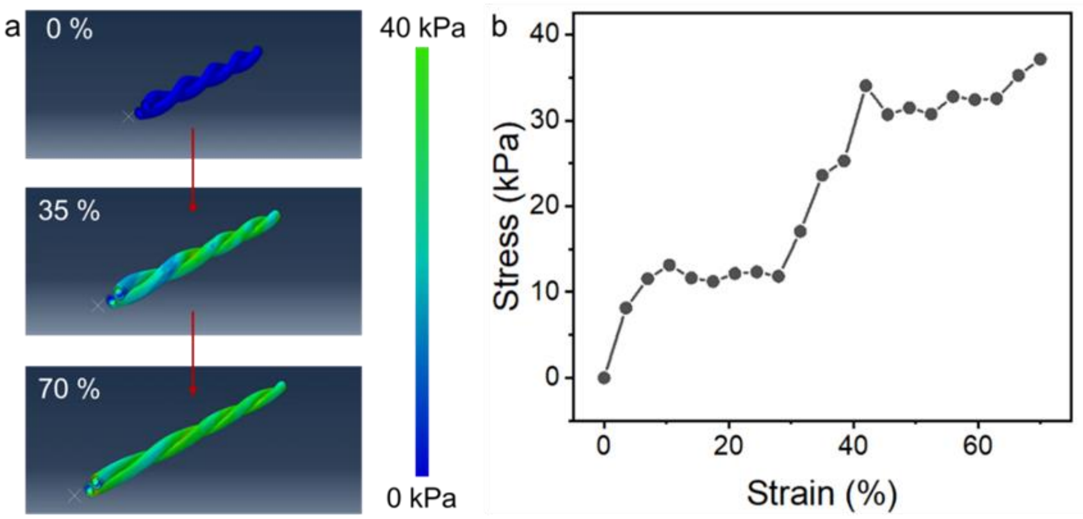

Figure S15. (a) Meshing and refinement of the simulation model on DPCSSYs. (b) The simulated stress generated at different strain levels.

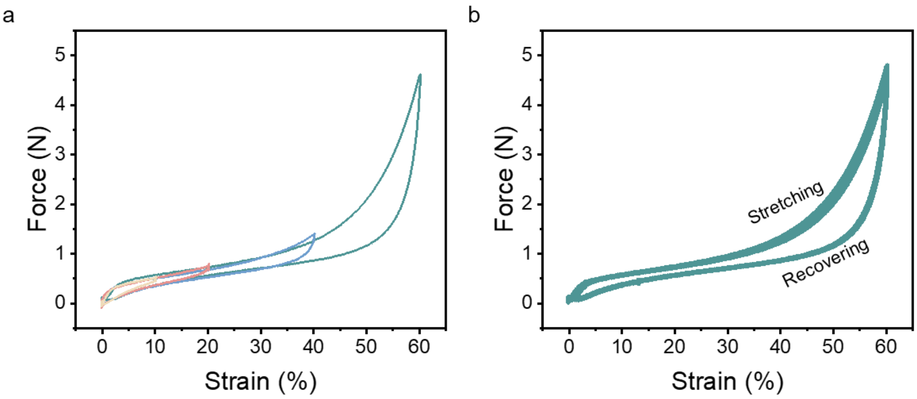

Figure S16. (a) Force-strain curves of the DPCCSYs under various strains, (b) Force-strain curves of the DPCCSYs under 60% strain for 100 stretching-releasing cycles.

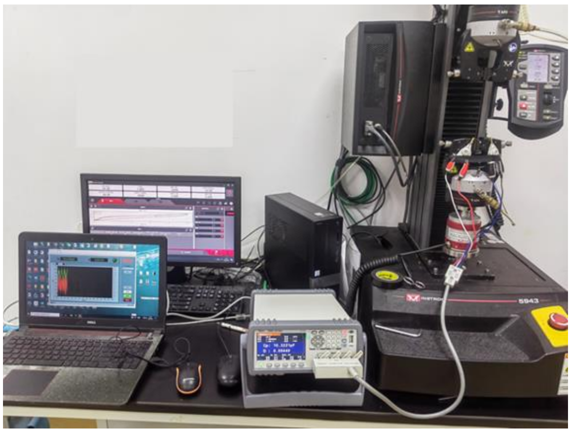

Figure S17. Testing system for sensing performance of DPCCSYs.

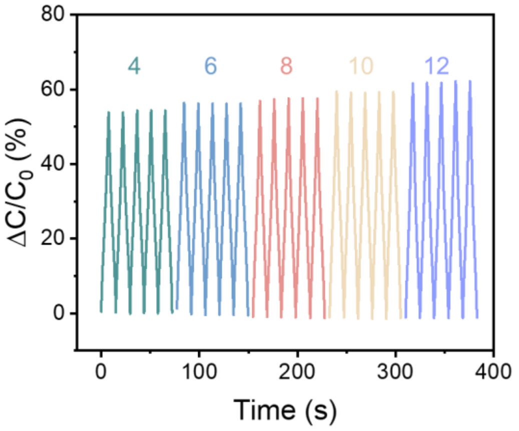

Figure S18. Relative capacitance changes of DPCSSYs with varied the number of core yarns.

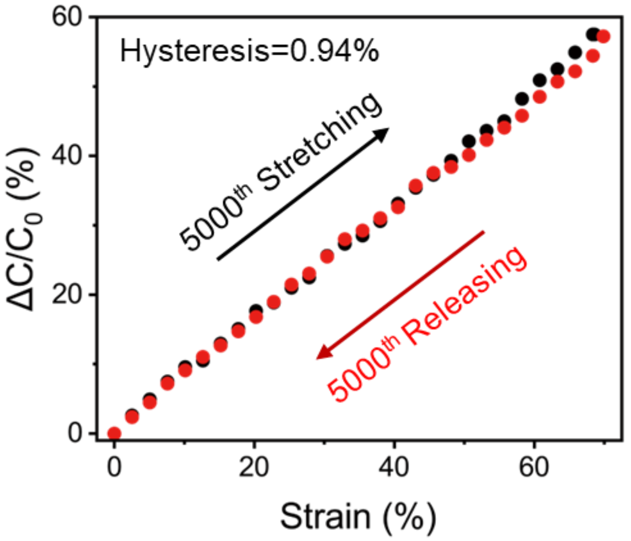

Figure S19. Hysteresis of DPCSSYs during 5000th stretching and releasing at 70 % strain.

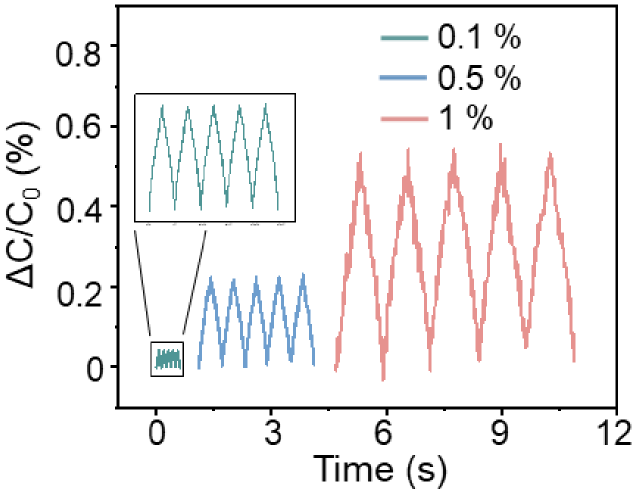

Figure S20. Relative capacitance changes of DPCSSYs under the strain of 0.1, 0.5 and 1%.

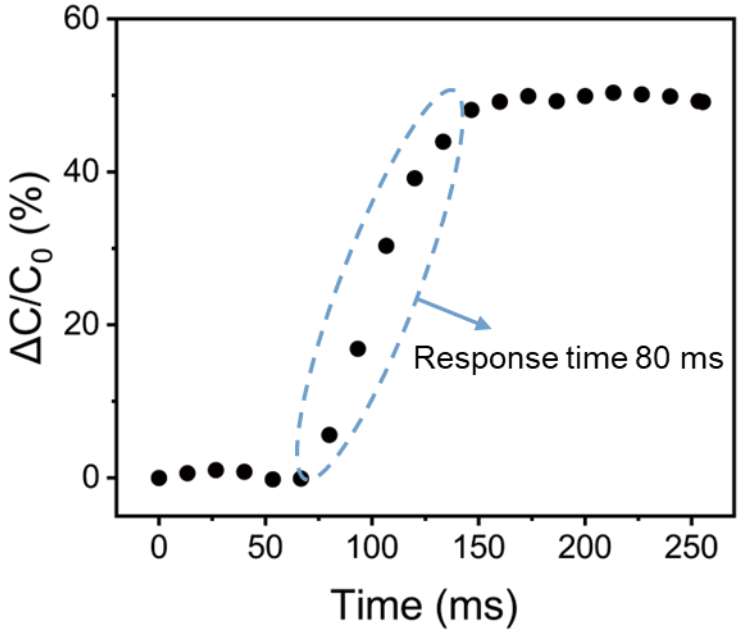

Figure S21. Response time of DPCSSYs at 50 % strain.

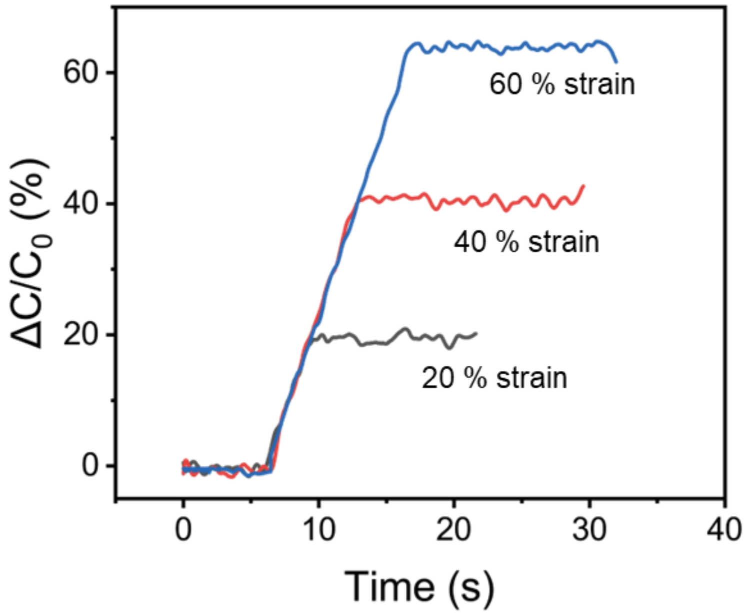

Figure S22. The ΔC C_0^{-1} change of DPCSSYs for a step strain of 20 %, 40 %, 60 %.

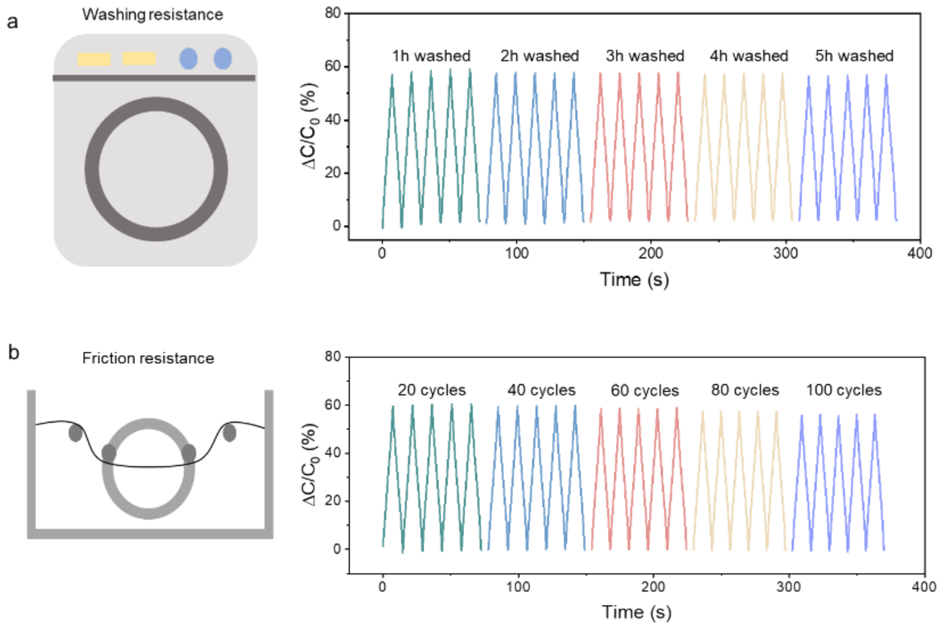

Figure S23. The $\Delta C/C_0$ of DPCCSYs after (a) washing and (b) abrasion tests.

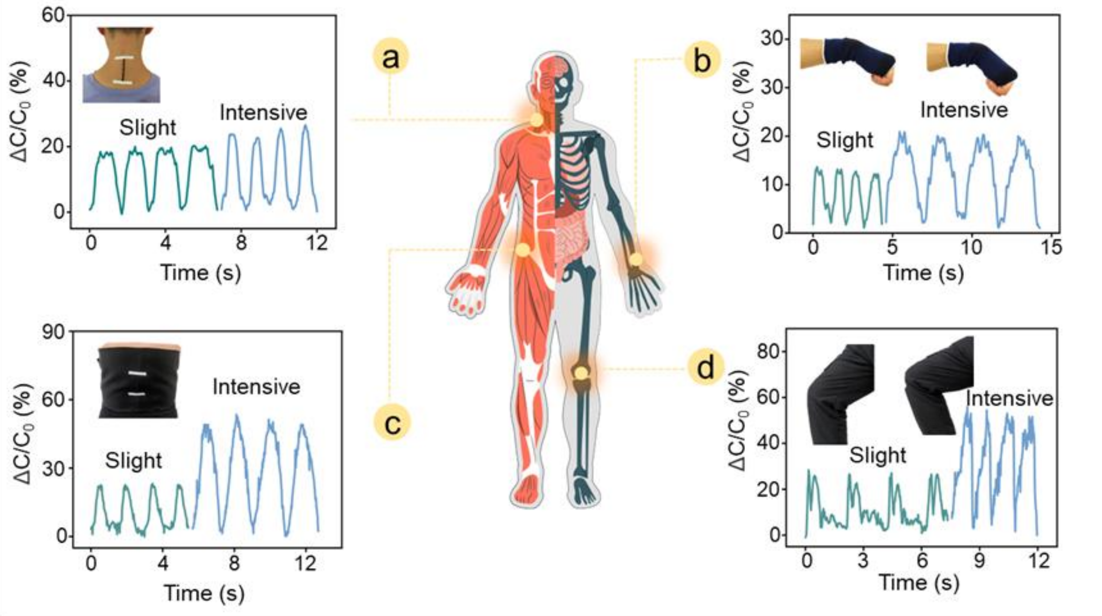

Figure S24. Application demonstrations of DPCSSYs in human motion monitoring.

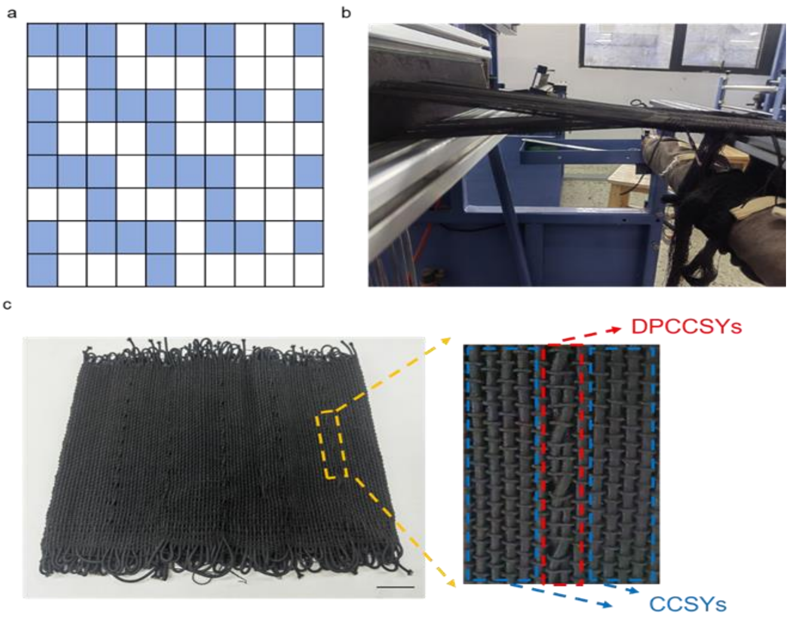

Figure S25. (a) Lifting plan and schematic illustration and (b) weaving process for the large-scale fabrication of the bifunctional smart textile. (c)The photographs of the bifunctional smart textile.

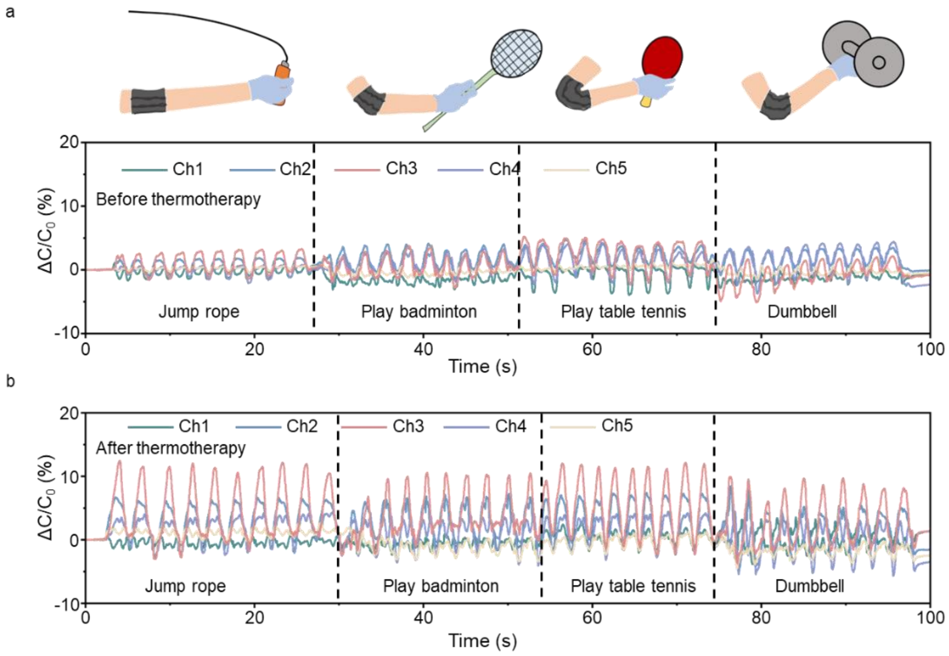

Figure S26. Multi-channel monitoring of various elbow movements with the bifunctional smart textile (a) before and (b) after thermotherapy.

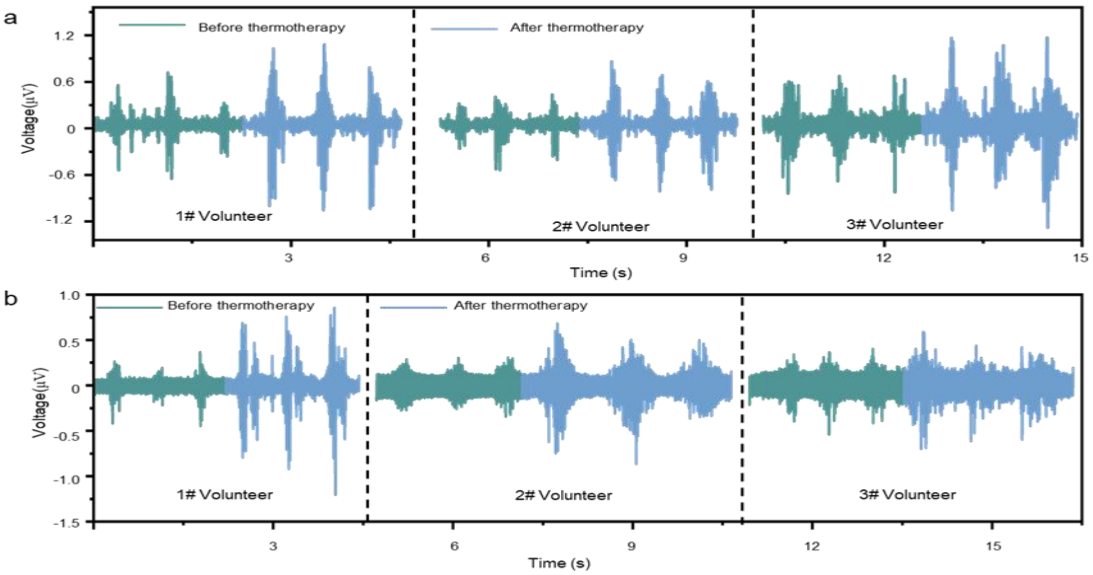

Figure S27. The EMG of the human (a) knee and (b) shoulder before and after thermotherapy.

	CCSYs textile	Cotton	Go-fabric	Mylar blanket
Thickness	~2.2 mm	~2.06 mm	~2.13 mm	~0.5 μm
Area density	~350 g/m^2	~310g/m^2	~335 g/m^2	~40 g/m^2

Table S1. The thickness and area density of different textiles.

Table S2. The comparison of sensing performance of CSSYs with reported flexible sensors.

Strain sensor materials	Structures	Sensing range (%)	Sensitivity (GF_{max})	Tested cycles [strain selection]	Ref.
AgNWs–Ecoflex	Sandwich	0−50	0.7	100	(1)
Carbon nanotubes on PDMS substrates	Films	0−50	0.004	2000	(2)
CNTs/PDMS	Sandwich	0-50	0.7	1000	(3)
Silver coated nylon fibers	Double-ply yarn	0−13	0.695	10000	(4)
Multi-walled carbon nanotubes (MCNTs)	Sandwich	0−100	0.5	2600	(5)
Multiwall carbon nanotube sheet (NTS)/ rubber fiber	Yarns	0−200	0.625	NA	(6)
CNT/PDMS	Three-dimensional	0-100	0.413	5000	(7)
AgNW/PU-Acrylic elastomeric dielectric spacer	NA	0-60	0.5	NA	(8)
**DPCSSYs**	**Core-shell, twisted yarn**	**0−70**	**0.854**	**5000**	**This work**

References:

(1) S. S. Yao, Y. Zhu, Nanoscale, **2014**, *6*, 2345.

(2) D. J. Lipomi, M. Vosgueritchian, B. C. Tee, S. L. Hellstrom, J. A. Lee, C. H. Fox, Z. Bao, Nat. Nanotechnol. **2011**, *6*, 788.

(3) H. Nesser, G. Lubineau, ACS Appl. Mater. Interfaces. **2021**, *13*, 36062.

(4) Q. Zhang, Y. L. Wang, Y. Xia, P. F. Zhang, T. V. Kirk, X. D. Chen, Adv. Mater. Technol. **2019**, *4*, 1900485.

(5) T.Y. Dong, Y. Gu, T. Liu, M. Pecht, Sensor Actuat. A-phys. **2021**, *326* ,112720.

(6) H.Y. Wang, Z.F. Liu, J.N. Ding, X. Lepró, S.L Fang, N. Jiang, N.Y. Yuan, R. Wang, Q. Yin, W. Lv, Z.S. Liu, M. Zhang, R. Ovalle-Robles, K. Inoue, S.G Yin, R. H. Baughman, Adv. Mater. **2016**, *28*, 4998.

(7) X.Y Wang, Y. Deng, P. Jiang, X.R Chen, H.Y. Yu, Microsyst. Nanoeng. **2022**, *8*, 113.

(8) W.L. Hu, X.F. Niu, R. Zhao, Q.B. Pei, Appl. Phys. Lett. **2013**, *102*, 083303.
